# Host association, environment, and geography underlie genomic differentiation in a major forest pest

**DOI:** 10.1111/eva.13466

**Published:** 2022-09-23

**Authors:** Zachary G. MacDonald, Kyle L. Snape, Amanda D. Roe, Felix A. H. Sperling

**Affiliations:** ^1^ Department of Biological Sciences University of Alberta Edmonton Alberta Canada; ^2^ UCLA La Kretz Center for California Conservation Science University of California Los Angeles Los Angeles California USA; ^3^ Institute of the Environmental and Sustainability University of California Los Angeles Los Angeles California USA; ^4^ Great Lakes Forestry Centre, Canadian Forest Service Natural Resources Canada Sault Ste. Marie Ontario Canada

**Keywords:** ecological selection, forest pest, host‐associated differentiation, isolation by ecology, isolation by environment, local adaptation, *Malacosoma*

## Abstract

Diverse geographic, environmental, and ecological factors affect gene flow and adaptive genomic variation within species. With recent advances in landscape ecological modelling and high‐throughput DNA sequencing, it is now possible to effectively quantify and partition their relative contributions. Here, we use landscape genomics to identify determinants of genomic differentiation in the forest tent caterpillar, *Malacosoma disstria*, a widespread and irruptive pest of numerous deciduous tree species in North America. We collected larvae from multiple populations across Eastern Canada, where the species experiences a diversity of environmental gradients and feeds on a number of different host tree species, including trembling aspen (*Populus tremuloides*), sugar maple (*Acer saccharum*), red oak (*Quercus rubra*), and white birch (*Betula papyrifera*). Using a combination of reciprocal causal modelling (RCM) and distance‐based redundancy analyses (dbRDA), we show that differentiation of thousands of genome‐wide single nucleotide polymorphisms (SNPs) among individuals is best explained by a combination of isolation by distance, isolation by environment (spatial variation in summer temperatures and length of the growing season), and differences in host association. Configuration of suitable habitat inferred from ecological niche models was not significantly related to genomic differentiation, suggesting that *M. disstria* dispersal is agnostic with respect to habitat quality. Although population structure was not discretely related to host association, our modelling framework provides the first molecular evidence of host‐associated differentiation in *M. disstria*, congruent with previous documentation of reduced growth and survival of larvae moved between natal host species. We conclude that ecologically mediated selection is contributing to variation within *M. disstria*, and that divergent adaptation related to both environmental conditions and host association should be considered in ongoing research and management of this important forest pest.

## 1 INTRODUCTION

Despite the considerable economic and ecological importance of forest pests, often little is known about the mechanisms that contribute to intraspecific variation (Gould, 2008; Janes et al., 2014; Lait & Hebert, 2018; Lumley et al., 2020; Nelson et al., 2022; Parry et al., 2001; Parry & Goyer, 2004; Rollins et al., 2006). The emerging field of landscape genomics provides a valuable toolkit for quantifying the relative contribution of biotic and abiotic factors to both neutral and adaptive differentiation, which can inform both ongoing research and applied management practices. In this study, we combine landscape and ecological modelling with high‐throughput DNA sequencing to identify factors that are contributing to genomic differentiation in an economically important and irruptive forest defoliator in North America.

Genomic differentiation can arise from a number of different mechanisms, often categorized as forms of geographic, environmental, or ecological isolation (MacDonald et al., 2020; Wang et al., 2013). The most common form of geographic isolation, isolation by distance (IBD), describes patterns in which genetic distance among individuals or populations positively correlates with their geographic separation (Wright, 1943). The frequent observation of IBD in nature is generally attributed to gene flow decreasing with increasing geographic distance, due to the limited dispersal ability of most organisms (Charlesworth et al., 2003; Meirmans, 2012; Petkova et al., 2016; Rousset, 1997; Slatkin, 1993; Vekemans & Hardy, 2004). An alternative model, isolation by resistance (IBR), builds on IBD and predicts that heterogeneous landscapes confer varying resistance to dispersal that affects gene flow (McRae, 2006; Zeller et al., 2012). Resistance surfaces can be parameterized as the inverse of predicted habitat suitability and used to estimate various least‐cost or resistance distances among individuals or populations, which may be used to assess IBR (Keeley et al., 2017; McDonald et al., 2020; McRae & Beier, 2007; Storfer et al., 2010; Wang et al., 2008; Wang et al., 2013; but see Peterman et al., 2014). Together, these forms of geographic isolation constitute an evolutionary null model, in which genomic differentiation arises in the absence of divergent selection (Coyne & Orr, 2004; Jenkins et al., 2010; MacDonald et al., 2020). In contrast, environmental/ecological isolation necessarily implicates divergent selection related to abiotic or biotic factors as the principal factor limiting gene flow and promoting genomic differentiation within species. Isolation by environment (Wang & Bradburd, 2014; Wang & Summers, 2010) and isolation by ecology (Claremont et al., 2011; Edelaar et al., 2012; Shafer & Wolf, 2013), which we collectively refer to as “IBE”, describe patterns in which genetic distance among individuals or populations positively correlates with environmental or ecological differences, independent of geographic isolation.

Spatially divergent adaptation to different environmental or ecological conditions is often cited as the principal mechanism underlying IBE (e.g., Coyne & Orr, 2004; Crispo et al., 2006; MacDonald et al., 2020; Sánchez‐Ramírez et al., 2018; Thorpe et al., 2008; Van Buskirk & van Rensburg, 2020; Wang et al., 2013). However, IBE can also operate in sympatry if populations are locally diverged in their respective niches (Nosil, 2012). A well‐studied example of IBE that may occur in sympatry is host‐associated differentiation (HAD) in phytophagous insects (Antwi et al., 2015; Berlocher & Feder, 2002; Bush, 1969; Drès & Mallet, 2002; Ehrlich & Raven, 1969; Jaenike, 1990; Leung & Beukeboom, 2021; Stireman et al., 2005; Vertacnik & Linnen, 2017). Within single species, different populations may specialize on different host plant species, with evolutionary divergences ranging from elevated levels of genomic differentiation to the initiation and reinforcement of speciation (Drès & Mallet, 2002; Driscoe et al., 2019; Forbes et al., 2017; Mackintosh et al., 2019; Medina, 2017; Peccoud et al., 2009). Mechanisms underlying this differentiation are generally hypothesized to involve divergent selection related to host detoxification and female oviposition preference (Birnbaum & Abbot, 2020; Cohen et al., 1992; Orsucci et al., 2018; Thompson & Pellmyr, 1991). Such relationships have been observed for sympatric populations in a number of insect taxa, e.g., *Eurosta solidaginis* (Waring et al., 1990), *Rhagoletis pomonella* (Feder et al., 2005), *Acyrthosiphon pisum* (Peccoud et al., 2009), and *Belonocnema treatae* (Driscoe et al., 2019). However, host specialization is frequently also observed among parapatric or allopatric populations, due in part to the close geographic association of phytophagous insects and their hosts and the possibility of range shifts/expansions made possible by host shifts (Hunter & Price, 1992; Jaenike, 1990; Underwood & Rausher, 2000). In these cases, inferring HAD can be difficult, because it is often unclear whether divergent host associations among parapatric or allopatric populations are a cause or consequence of geographic separation (Forbes et al., 2017; Hunter & Price, 1992; Lancaster, 2020; Singer & Parmesan, 2021; Underwood & Rausher, 2000). Regardless of the geographic mode of differentiation, landscape genomic analyses make it possible to control for spatial confounds and partition genomic differentiation into geographic and environmental/ecological components, including host association (Driscoe et al., 2019; Legendre et al., 2015; Legendre & Fortin, 1989; Shafer & Wolf, 2013).

In this study, we use landscape genomics to identify biotic and abiotic factors that best explain genomic differentiation within the forest tent caterpillar, *Malacosoma disstria* Hübner. Specifically, we quantify and compare the relative effects of geographic isolation (IBD + IBR), variation in environmental conditions (IBE), and host association (HAD as a special form of IBE) on population structure and genomic differentiation. *Malacosoma disstria* larvae are important and irruptive forest defoliators in North America, known to feed on at least 15 plant species (Charbonneau et al., 2012; Fitzgerald, 1995; Hartmann & Messier, 2011; Parry & Goyer, 2004). Despite the considerable economic and ecological importance of this species, and documented functional differences in‐based larval growth and survival linked to host association, few studies have investigated geographic, environmental, or ecological determinants of intraspecific variation. One study, addressing mitochondrial DNA, suggested that complex phylogeographic patterns observed across the species' range were due to geographic isolation during Pleistocene glaciations and post‐glacial dispersal (Lait & Hebert, 2018). However, complex population structure at finer spatial scales remains unexplained, and no attempt has been made to link genomic differentiation with sympatric differences in host association.

To evaluate the relative contributions of IBD, IBR, and IBE/HAD in *M. disstria*, we quantified genomic variation using thousands of genome‐wide single nucleotide polymorphisms (SNPs) generated from reduced‐representation sequencing of 159 larvae collected from four different host tree species in Eastern Canada. Our landscape analyses implemented a series of ecological niche models to map suitable habitat within the study area and generate a series of resistance distance measures between sequenced individuals. These measures were then contrasted with environmental and ecological distance measures, including differences in local environmental conditions and host association, to partition genomic variation into geographic (IBD + IBR) and environmental/ecological (IBE + HAD) components. Finally, we employed a combination of genotype‐environment association and *F*_ST_‐based outlier analyses to assess whether our reduced‐representation SNP dataset included genomic regions that may be under divergent selection related to variation in ecological/environmental conditions or host association.

## 2 MATERIALS AND METHODS

### 2.1 Sample collection

**FIGURE 1:**
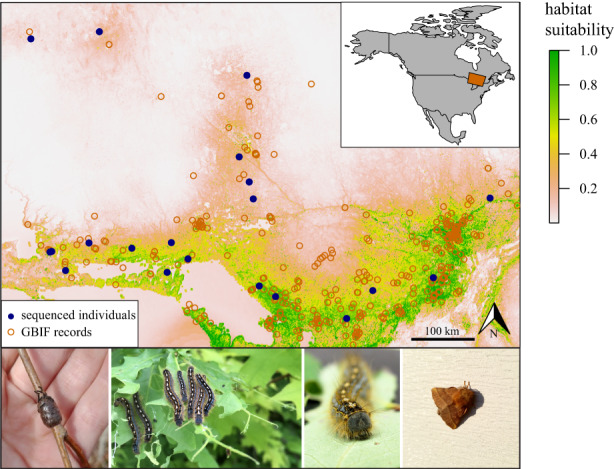
Map of the study area where *Malacosoma disstria* egg bands and larvae were sampled (*n* = 21 collection locations). Individuals were collected from four host tree species: Trembling aspen (*Populus tremuloides*), sugar maple (*Acer saccharum*), red oak (*Quercus rubra*), and white birch (*Betula papyrifera*). Ecological niche models, parameterized using the collection locations of sequenced individuals and *M. disstria* global biodiversity information facility (GBIF) records, were used to predict habitat suitability across the study area. Predictors included both geographic and environmental/ecological geographic information system (GIS) variables. Within the map of predicted habitat suitability, higher index scores correspond to higher suitability.

Our study focused on *M. disstria* populations across Eastern Canada (Figure 1), where the species experiences a diversity of environmental gradients and feeds primarily on trembling aspen (*Populus tremuloides*), sugar maple (*Acer saccharum*), red oak (*Quercus rubra*), and white birch (*Betula papyrifera*). *Malacosoma disstria* females lay a single egg band and emerging larvae form a cohesive family group (McClure & Despland, 2010). The gregarious larvae remain on their natal host for the first 4–5 instars (Batzer et al., 1995; Schowalter, 2017), taking 22–45 days depending on local environmental conditions (Witter & Kulman, 1972). Our field sampling took place in 2018 and consisted of collecting egg bands or larvae from family groups from separate trees, aiming for 10–20 different samples per collection location, distributed across as many host tree species as possible within each site. Site descriptions and local outbreak conditions based on aerial defoliation surveys are described in Table S1. Egg bands and larvae were transported live to the Insect Production and Quarantine Laboratories (IPQL) facility at the Great Lakes Forestry Centre in Sault Ste. Marie, Ontario. We reared each family unit separately until larvae reached 3rd to 5th instars on fresh, locally collected foliage of their recorded host tree species. Rearing conditions were constant at 27°C, 55% R.H., and 16:8 h light:dark in accordance with the IPQL rearing protocol for *M. disstria*. We subsampled and preserved 1st and 2nd instars from each family group in case disease or death occurred. To euthanize and preserve larvae, individuals were placed in 95% ethanol and frozen at −20°C. We also continued to rear a subset of larvae from each collection location to adult, at which point individuals were frozen and preserved at −20°C.

### 2.2 DNA extraction and sequencing

Specimen preparation differed between larval and adult individuals. For larvae, we dissected tissues from the head capsule and upper thorax, removing the digestive tract to reduce the probability of host plant or microbial contamination (*n* = 104). Thoracic tissue was dissected from adult individuals (*n* = 45). We extracted genomic DNA from these tissues using the Qiagen DNeasy Blood and Tissue Extraction kit (QIAGEN), following the manufacturer's protocol with the addition of bovine pancreatic ribonuclease A treatment (RNaseA, 4 μl at 100 mg/ml; Sigma‐Aldrich Canada Co.). Following extraction, genomic DNA was ethanol precipitated and stored in 50 μl Millipore water at −20°C. We prepared double‐digest restriction site‐associated DNA sequencing (ddRADseq) libraries from 200 ng genomic DNA using *MspI* and *PstI* restriction enzymes, following the protocol of MacDonald et al. (2020). In general, wet lab and Illumina adapter dual indexing procedures were modified from Poland et al. (2012) and Peterson et al. (2012), respectively. A pooled library of 149 individually indexed samples was sequenced at the University of Alberta's Molecular Biology Services Unit using single‐end, 75‐bp sequencing on a single high output flowcell of an Illumina NextSeq 500.

### 2.3 Bioinformatic processing

We used “process_radtags” in the program Stacks 2.0 (Rochette et al., 2019) to demultiplex Illumina single‐end, 75‐bp reads. Reads with quality scores below 20 within a sliding window 15% of the read length were filtered from the dataset. Illumina index sequences (8 bp) were identified and removed (with one mismatch permitted), resulting in 67‐bp reads. We then used the program Cutadapt 1.9.1 (Martin, 2011) to identify and remove remnant Illumina adaptor sequences and remove the first 5 bp from the 5′ end of each read corresponding to the *PstI* restriction site. The 62‐bp reads were finally aligned to a *M. disstria* genome assembly (NCBI Accession PRJNA824522) comprised of 1090 scaffolds (each over 10 kb) using Burrows–Wheeler Aligner 0.7.17 (BWA‐MEM) (Li, 2013; Li & Durbin, 2009). Resulting SAM files were converted to BAM format using SAMtools 1.9 (Li et al., 2009). We then used “gstacks” and “populations” within Stacks 2.0 to call SNPs, stipulating a single population. We filtered the resulting dataset using VCFtools 0.1.14 (Danecek et al., 2011), removing: (1) individuals with more than 25% missing data; (2) loci with read depths less than five; (3) loci with minor allele frequencies less than 0.05; (4) loci with percentages of missing data greater than 5%; and (5) one locus for every pair of loci that were within <10 kb of each other. This 10 kb thinning interval was based on distances in which linkage disequilibrium (LD) has been documented to decay within other Lepidoptera; for example, LD decays to baseline within 1–10 kb in *Heliconius* spp. (Martin et al., 2013) and 100 bp in *Danaus plexippus* (Zhan et al., 2014); LD decays to half of maximum within 7–46 bp in *Bombyx mori* (Xia et al., 2009) and 200 bp in *Helicoverpa armigera* (Song et al., 2015).

In a number of cases, we extracted and sequenced genomic DNA from multiple larvae reared from single egg bands, meaning full‐sibling relationships were expected and could bias population genomic analyses (O'Connell et al., 2019). To identify putative full siblings, we used the R package SNPRelate v. 1.26.0 (Zheng et al., 2012) to estimate pairwise kinship coefficients among all sequenced individuals. For diploid organisms, the coefficient value expected for full siblings is 0.25. Among all sequenced individuals, a natural break in coefficient values occurred at 0.22; values above which were only observed for pairs of individuals collected from the same location. For each pair of putative full siblings, we removed the individual with the greater percentage of missing data (*n* = 37). We then reverted to original BAM files, recalled SNPs, and repeated filtering according to the parameters specified above.

### 2.4 Population genetic structure

To visualize population structure with no a priori expectations of clustering, we performed principal component analysis (PCA) on genomic data using the R package adegenet v. 2.1.1 (Jombart, 2008). Next, to visualize host‐associated genomic divergence among *M. disstria* individuals, we completed discriminant analysis of principal components (DAPC; Jombart et al., 2010) using host tree species as the a priori grouping. The “xvalDapc” function (adegenet package; 100 replicates) was used to estimate the optimal number of PCs to retain in DAPC using stratified cross‐validation. Missing genotypes were imputed as locus means for cross‐validation. Visualizing DAPC allowed us to subjectively infer whether there was substantial genomic separation of individuals based on their host tree species. Finally, we used the model‐based clustering program *structure* 2.3.4 (Pritchard et al., 2000) to infer the optimal value of *K* and assign individuals to discrete clusters based on admixture coefficients. Ten independent runs were completed for each value of *K* = 1:10 using the admixture model and correlated allele frequencies. The burn‐in period and number of Markov chain Monte Carlo (MCMC) repetitions were set to 100,000 and 1,000,000, respectively. Location prior values (*locprior* parameter) were set to collection localities (*n* = 21) to inform the MCMC algorithm without biasing the model. The alpha prior (relative admixture levels between populations) was set to 0.25; equal to one divided by the number of host tree species (*n* = 4), we collected individuals from (Wang, 2017).

### 2.5 Habitat suitability

We predicted and mapped habitat suitability for *M. disstria* within the study area using ecological niche models generated with MaxEnt software (Phillips et al., 2006) implemented via the R package dismo v. 1.3–3 (Hijmans et al., 2011). Briefly, MaxEnt uses machine learning maximum entropy modelling to infer habitat suitability using presence‐only species records and geographic information systems (GIS) predictor variables. Presence‐only records included in our MaxEnt models included both the collection locations of sequenced individuals and georeferenced *M. disstria* occurrences downloaded from the Global Biodiversity Information Facility (GBIF; accessed November 4, 2020). We generated a 50‐km minimum convex polygon around the collection locations of our sequenced individuals to define the study area (Fourcade et al., 2014; Phillips & Dudík, 2008) and cropped GBIF occurrence records to this polygon (*n* = 760). All duplicate localities were removed, resulting in 595 unique occurrence records (sequenced individuals + GBIF records). Geographic predictor variables included terrain ruggedness, heat load (based on terrain slope and aspect), and land cover (12 categories). Environmental predictor variables included mean temperature of the warmest month, mean temperature of the coldest month, the difference between mean temperatures of the warmest and coldest months (hereafter, “continentality”), degree days below 0°C (chilling degree days), degree days above 5°C (growing degree days), extreme minimum temperature, mean summer (June to August) precipitation, and length of the frost‐free period. Terrain ruggedness and heat load indices were calculated using the R packages raster (Hijmans, 2021) and spatialEco (Evans, 2021), respectively, using a digital elevation model (Wang et al., 2016). Land cover GIS data were acquired from the Commission for Environmental Cooperation (http://www.cec.org/) and generated using 2015 Landsat satellite imagery. Environmental and elevation GIS data were compiled using ClimateNA v5.10 software (Wang et al., 2016). Each GIS data layer was reprojected to an equal‐area projection (Lambert Conformal Conic) at 1‐km resolution.

We ran MaxEnt models using 10,000 background points to sample available habitat, making all feature classes available and setting the regularization parameter to 1.0 (Phillips, 2005). To evaluate predicative power, we withheld 20% of occurrence localities for cross‐validation and receiver operating characteristic (ROC) analysis using five different models (Phillips et al., 2006). We then averaged these five models to predict habitat suitability (logistic output) across the study landscape using the “predict” function (raster package). All 1‐km grid cells within the study area received a predicted habitat suitability score ranging from 0 to 1, with higher values indicating higher suitability.

### 2.6 Geographic and environmental/ecological distances

We estimated geographic distances between all sequenced individuals using three measures; Euclidean distance, least‐cost distance, and resistance distance. Euclidean distance represents the minimum distance that a dispersing individual is required to travel between two locations regardless of landscape characteristics. We calculated Euclidean distances among all sequenced individuals using the “spDists” function in the R package “sp” (Pebesma & Bivand, 2005) and organized them into a pairwise distance matrix. In contrast to Euclidean distance, least‐cost and resistance distances account for the relative resistance organism are hypothesized to experience while moving across landscapes. Least‐cost distances are estimated by searching for single, optimal routes across resistance surfaces, and thereby assume that organisms have complete knowledge of landscapes before dispersal. In contrast, resistance distances (analogous to circuit distances) consider a multitude of possible paths based on random walks (or circuit theory), with greater cumulative resistance between two points amounting to greater a distance value (McRae & Beier, 2007). Using a resistance surface parameterized as the inverse of predicted habitat suitability, we estimated pairwise least‐cost and resistance distances using the R package gdistance (van Etten, 2018). This method of parametrization effectively tests the hypothesis that individuals are more likely to disperse within suitable habitat and experience greater resistance when moving through unsuitable habitat (Keeley et al., 2017; MacDonald et al., 2020; McRae & Beier, 2007; McRae, 2006; Sánchez‐Ramírez et al., 2018; Storfer et al., 2010; Thorpe et al., 2008; Wang et al., 2008; Wang et al., 2013).

We estimated environmental/ecological distances between all sequenced individuals using the same environmental variables included in our MaxEnt model, taking the absolute difference of each variable's values at each individual's collection location (MacDonald et al., 2020; Wang et al., 2013). To quantify HAD, we generated a single, binary host association distance. For each pair of individuals, this host association distance quantified whether individuals were collected on the same (0) or different (1) host tree species. Each of these environmental/ecological distances was organized into pairwise matrices, commensurate with the geographic distance matrices generated above.

### 2.7 Determinants of genomic differentiation

We used two different modelling methods to infer the effects of geographic isolation (IBD + IBR), variation in environmental conditions (IBE), and host association (HAD as a special form of IBE) on genomic differentiation within *M. disstria*. The first method, reciprocal causal modelling (RCM), compares relative support between pairs of geographic, environmental, or ecological distances, allowing us to infer which variables best explain variation in genomic differentiation after controlling for the effects of all others. The second, distance‐based redundancy analysis (dbRDA), partitions variation in genomic differentiation among multiple predictor variables, allowing us to simultaneously evaluate contributions of geographic isolation, variation in environmental conditions, and host association. For both analyses, genomic differentiation among sequenced individuals was estimated as pairwise Euclidean genetic distance using the “dist” function within the R package adegenet (sum of squared Euclidean distances between *i* ^th^ and the *j* ^th^ genotype). This simple distance measure has been shown to effectively quantify within‐species genomic variation in both simulations (Shirk et al., 2017) and empirical research (MacDonald et al., 2020; Sánchez‐Ramírez et al., 2018).

#### 2.7.1 Reciprocal causal modelling

Our first analysis implemented RCM with partial Mantel tests (Cushman et al., 2006, 2013). Each reciprocal model was composed of two partial Mantel tests (999 permutations), completed using R package vegan (Oksanen et al., 2007), for a total of 132 tests organized into 66 reciprocal models. Within each reciprocal model, partial Mantel test A estimated the partial Mantel's *R* coefficient “*R*_*PM‐A*_" between genetic distance and one of two geographic, environmental, or ecological distances (focal variable) conditioned on the other distance (alternative variable). For partial Mantel test B, the focal and alternative variables were reversed, producing partial Mantel's *R* coefficient “*R*_*PM‐B*_". If *R*_*PM‐A*_ > *R*_*PM‐B*_, the focal variable from partial Mantel test A is better supported. Conversely, if *R*_*PM‐A*_ < *R*_*PM‐B*_, the alternative variable from partial Mantel test A is better supported. A simple index, estimated as *R*_*PM‐A*_–*R*_*PM‐B*_, quantifies relative support among the two variables involved in one reciprocal model. To visualize these results, we summarized index values using a heatmap, wherein variables with more warm colors in their rows are better supported.

#### 2.7.2 Distance‐based redundancy analysis

To simultaneously investigate and partition the effects of geographic isolation, variation in environmental conditions, and host association on genomic differentiation, we used dbRDA using genetic distance as the response matrix (Legendre & Anderson, 1999). In this analysis, geographic isolation (IBD) and spatial autocorrelation of allele frequencies were accounted for using distance‐based Moran's eigenvector mapping (dbMEM), synonymous with the principal coordinates of neighbor matrices (PCNM) method described by Borcard and Legendre (2002) (e.g., Driscoe et al., 2019; Jardim de Queiroz et al., 2017; Mikheyev et al., 2013). We constructed individual‐based dbMEM variables using the pairwise matrix of Euclidean distances among all sequenced individuals. As a first step, we generated a minimum spanning tree to identify a minimum spanning distance, equal to the maximum nearest‐neighbor distance across all pairs of individuals (Borcard et al., 2004). All pairs of individuals were next categorized as either “neighbors” or “not neighbors” based on a threshold distance of 259.81 km, equal to four times the calculated minimum spanning distance of 64.95 km. Pairwise Euclidean distances greater than this threshold (i.e., “not neighbors”) were truncated to 259.81 km. We then completed dbMEM using the “pcnm” function with the R package vegan (Oksanen et al., 2007), which applies principal coordinate analysis (PCoA) on the truncated Euclidean distance matrix. Positive eigenvectors (*n* = 11) were then extracted from PCoA as individual‐based geographic distance measures.

We attained a parsimonious dbRDA model by first applying dbRDA on genetic distance using the 11 positive eigenvectors as predictors and only retaining eigenvectors with significant effects (e.g., Driscoe et al., 2019). Next, we reduced collinearity among environmental predictors by performing PCA on the eight environmental rasters using the “rasterPCA” function from the R package “RStoolbox” (Leutner et al., 2019). We extracted PCs that explained >1% of the total variance in the data and included the corresponding PC rasters as environmental predictor variables by extracting values at the collection locations of all sequenced individuals. This resulted in a reduced set of environmental variables that effectively measured variation in environmental conditions among sampling locations while minimizing collinearity and model complexity. Host tree species was also included as a predictor in the final dbRDA model. All non‐categorical variables were standardized (subtracting the mean and dividing by standard deviation), permitting comparisons of effect sizes. The significance of each predictor variable was evaluated using permutational ANOVA applied to dbRDA. Finally, we used the “varpart” function (vegan package) to partition variation in genomic differentiation among geographic, environmental, and host association predictor variables.

### 2.8 Host‐associated divergent selection

We grouped all sequenced individuals according to their host tree species and examined divergence on a locus‐by‐locus basis to assess whether specific genomic regions are associated with HAD. Specifically, we used BayeScan 2.1 (Foll & Gaggiotti, 2008) to identify *F*_ST_ outlier loci that indicate divergent selection. BayeScan is recognized as an effective method for identifying outlier loci when discrete groupings of individuals are sensible (De Mita et al., 2013; Lotterhos & Whitlock, 2014; Narum & Hess, 2011). Host groups were organized into six pairwise comparisons for *F*_ST_ estimation of all loci. For each of the six pairs, we re‐filtered the genomic data for all individuals using the protocol described above, ensuring all SNPs were biallelic. We then completed 15 BayeScan runs using the following settings: prior odds = 10, thinning interval = 10, number of pilot runs = 20, length of pilot runs = 5000, burn‐in length = 50,000, and number of outputted iterations = 10,000. The significance of *F*_ST_ outliers was assessed using *q*‐values according to the False Discovery Rate (FDR) criterion (Benjamini & Hochberg, 1995) and an *α*‐threshold of 0.05.

### 2.9 Environmental associations of individual loci (LFMM)

If IBE is detected within species, it can be inferred that local adaptation to environmental/ecological conditions is an important mechanism limiting gene flow and promoting genomic differentiation (Edelaar & Bolnick, 2012; Hendry, 2004; MacDonald et al., 2020; Wang & Bradburd, 2014). Genotype‐environment analyses are a powerful tool for identifying specific genomic regions that may be linked to traits under selection (Rellstab et al., 2015). To assess whether our SNP dataset included any such regions, we used Latent Factor Mixed Modelling (LFMM) v. 1.3 (Frichot et al., 2013) and LFMM2 (Caye et al., 2019) implemented via the R package LEA (Frichot & François, 2015). Both methods correlate allele frequencies with continuous environmental variables on a locus‐by‐locus basis while controlling for background population structure. This is accomplished using latent factors equal in number to the optimal value of *K*, which we inferred here from *structure* analyses. Inclusion of latent factors reduces the likelihood of resolving spurious genotype‐environment relationships due to autocorrelation of space, population structure/demography, and environmental variables (Frichot et al., 2013). LFMM and LFMM2 differ in their methods of assessing correlations between allele frequencies and environmental variables, with LFMM implementing a Bayesian approach (Gibbs sampling algorithm) and LFMM2 regularized least‐squares minimization in latent factor regression models. While LFMM2 is faster and more conservative (a lower likelihood of false positives), LFMM has been shown to exhibit greater power to resolve significant genotype‐environment associations for small datasets (common for ddRADseq), when the specified value of *K* is low or when population structure is continuous (Luo et al., 2021).

We completed LFMM and LFMM2 analyses using the same eight environmental variables used in the analyses detailed above. For LFMM, we completed five runs per variable, each composed of 10,000 iterations and a burn‐in of 5000, and calculated the median |*z*|‐score across runs to infer the strength of each environmental association for each locus. To validate the number of latent factors used, we first estimated adjusted *p*‐values using the genomic inflation factor (λ) procedure (Devlin & Roeder, 1999) and then inspected their distribution for each environmental variable. Relatively flat distributions with a peak near zero indicate that the number of latent factors is sufficient (Frichot & François, 2015). We controlled for multiple tests both by applying a Bonferroni correction to adjusted *p*‐values and using the FDR criterion (Benjamini & Hochberg, 1995). Due to collinearity among environmental variables, single loci may exhibit significant associations with more than one environmental variable. In these cases, we identified the strongest association based on median |*z*|‐scores to determine which variable is most likely implicated in ecologically mediated selection (De Kort et al., 2015; MacDonald et al., 2020).

## 3 RESULTS

### 3.1 Genomic data

A total of 162,409,234 reads were sequenced and passed Illumina quality filters across all 149 sequenced individuals. After running “process_radtags” and associated filters, 158,059,066 reads were aligned to the *M. disstria* reference genome. Removing individuals with >25% missing data (*n* = 7) and putative full siblings (*n* = 38), and filtering loci based on read depth, minor allele frequency, missing data, and physical proximity, resulted in a final genomic dataset of 3114 SNPs with a mean read depth of 26.95 for 104 individuals.

### 3.2 Population genetic structure

**FIGURE 2:**
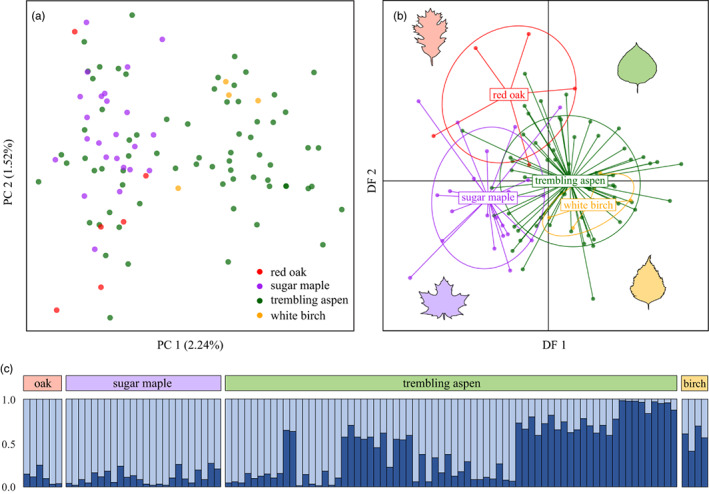
Population genetic structure of *Malacosoma disstria*, using (a) principal component analysis (PCA), (b) discriminant analysis of principal components (DAPC), and (c) model‐based clustering with *structure*. For PCA and DAPC plots, every point represents a sequenced individual (*n* = 104), color coded according to the host tree species it was collected from. Our *structure* analyses addressing *K* = 1:10 found an optimal value of *K* = 2; plotted bars show each individual's proportional membership to each cluster. Within the admixture plot, individuals are sorted according to host association and then by increasing latitude.

Visualization of clustering within PCA suggested that genomic variation in *M. disstria* is not discretely structured by host association (Figure 2). Some degree of separation was evident in DAPC when individuals were assigned to a priori groupings according to their host tree species. However, these ordination methods cannot resolve whether HAD is a mechanism underlying genomic differentiation, or is merely correlated with other causal environmental/ecological factors. Our *structure* analyses addressing *K* = 1:10 predicted an optimal value of *K* = 2 using both the Δ*K* method (Evanno et al., 2005) and the rate of change in the likelihood of *K* from 1:10 (Pritchard et al., 2000; see Figures S1 and S2). Assignments of individuals to population clusters did not accord with host association for any value of *K*, and individuals collected from different host species did not fall into discrete groups.

### 3.3 Habitat suitability

Our MaxEnt models predicted habitat suitability across the study landscape (Figure 1) with a high degree of accuracy, indicated by a mean AUC score of 0.88 (min = 0.86, max = 0.91). The relative contributions of each variable were estimated as mean permutational importance based on AUC values, reported here in descending importance: growing degree days = 39.86 (SE = 4.83), length of the frost‐free period = 17.61 (SE = 5.06), terrain ruggedness = 11.96 (SE = 2.85), continentality = 7.57 (SE = 1.84), extreme minimum temperature = 6.57 (SE = 8.51), chilling degree days = 4.87 (SE = 2.58), land cover = 3.95 (SE = 0.95), mean temperature of the warmest month = 3.46 (SE = 0.69), mean summer precipitation = 3.35 (SE = 1.29), mean temperature of the coldest month = 0.75 (SE = 0.74), and heat load = 0.06 (SE = 0.03). The inverse of predicted habitat suitability was used to parameterize a resistance surface to estimate pairwise least‐cost and resistance distances among the collection locations of sequenced individuals.

### 3.4 Determinants of genomic differentiation

#### 3.4.1 Reciprocal causal modelling

**FIGURE 3:**
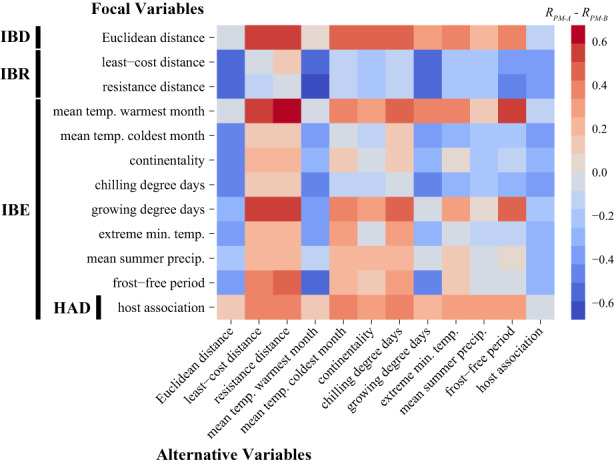
Pairwise heatmap visualizing results of reciprocal causal modelling (RCM) that assess correlates of genomic differentiation in *Malacosoma disstria*. Variables included in this analysis were categorized as measures of isolation by distance (IBD), isolation by resistance (IBR), isolation by environment/ecology (IBE), or host‐associated differentiation (HAD as a form of IBE). Euclidean distances were estimated between all sequenced individuals. Least‐cost and resistance distances were estimated using a resistance surface parameterized as the inverse of predicted habitat suitability. Environmental/ecological distances were measured as the absolute difference in the values of environmental variables at the collection location of sequenced individuals. Host association measured whether sequenced individuals were collected on the same (0) or different (1) host tree species. Within the heatmap, values in each cell represent results of *R*_*PM‐A*_–*R*_*PM‐B*_, with red and blue colors indicating positive and negative values, respectively. Rows and columns contain the focal and alternative variables, respectively, for partial Mantel test A within each reciprocal model. This heatmap should be interpreted by rows and not columns; variables on the *y*‐axis with more positive (red) values in their rows are the strongest correlates of genomic differentiation after partialling out relationships with alternative variables.

We summarized support for the effects of IBD, IBR, IBE, and HAD on genomic differentiation in RCM analysis using a heatmap (Figure 3), with red and blue colors indicating positive and negative values for *R*_*PM‐A*_–*R*_*PM‐B*_, respectively (see Table S3 for *R*_*PM‐A*_–*R*_*PM‐B*_ values and Table S4 for *p*‐values of *R*_*PM‐A*_). For each reciprocal model, the focal variable from partial Mantel test A is reported on the *Y*‐axis and the alternative variable on the *X*‐axis. This heatmap is best interpreted by focusing on rows; focal variables with more warm colors (higher index values) in their rows are more supported. Overall, the strongest correlate of genetic distance after partialling out alternative variables was host association, followed by Euclidean distance, difference in the mean temperature of the warmest month, and difference in growing degree days. This suggests that both geographic and environmental/ecological factors, including host association, have significant effects on genomic differentiation within *M. disstria*.

#### 3.4.2 Distance‐based redundancy analysis

We used a series of analyses to construct a dbRDA model that effectively quantified and partitioned the effects of geographic isolation (IBD), variation in environmental conditions (IBE), and host association (HAD) on genomic differentiation in *M. disstria*. First, we completed a preliminary spatial dbRDA using dbMEM positive eigenvectors as predictor variables (*n* = 11). Permutational ANOVA applied to dbRDA identified four dbMEM variables that were significantly related to genetic distance at *α* = 0.05 (dbMEMs 1, 2, 3, and 6; Table S5). Next, in a PCA of eight environmental variables, three PCs explained more than 1% of the total variance in environmental conditions. Values extracted from the corresponding PC rasters were used as composite measures of environmental variation in dbRDA. The final dbRDA model, therefore, consisted of four dbMEM variables (representing IBD), three composite environmental variables (representing IBE), and host association (representing HAD). All variance inflation factors were less than 10.

Our final dbRDA model explained 10.87% of genomic differentiation in *M. disstria*. Permutational ANOVA applied to dbRDA resolved that IBD, IBE, and HAD each had significant effects (Table 1). Significant predictor variables included the second dbMEM variable (i.e., the second positive eigenvector; *p* = 0.032), all three composite environmental variables (PCs 1, 2, and 3; *p* = 0.001, *p* = 0.041, and *p* = 0.001, respectively), and host association (*p* < 0.001). Overall, geographic isolation (IBD) accounted for 3.93% of genomic differentiation in *M. disstria*, variation in environmental conditions (IBE) accounted for 3.74%, and host association (HAD) accounted for 3.20%.

**TABLE 1:** Significance of geographic, environmental, and host association predictor variables in dbRDA analysis determined using permutational ANOVA

	*df*	Sum Of Sqs	*F*	*p*‐Value
dbMEM 1	1	0.473	1.021	0.119
dbMEM 2	1	0.479	1.035	**0.032**
dbMEM 3	1	0.455	0.982	0.863
dbMEM 6	1	0.468	1.011	0.253
environmental PC 1	1	0.62	1.340	**0.001**
environmental PC 2	1	0.478	1.032	**0.047**
environmental PC 3	1	0.505	1.090	**0.001**
host association	3	1.468	1.058	**0.001**

*Note*: Geographic variables are positive eigenvectors from distance‐based Moran's eigenvector mapping, including dbMEMs 1, 2, 3, and 6. Environmental variables are the first three principal components extracted from PCA applied to eight environmental variables. Host association is a categorical variable reflecting the host tree species from which each sequenced individual was collected. Significant *p*‐values (*α* = 0.05) are highlighted in bold text.

### 3.5 Host‐associated divergent selection

We grouped all sequenced individuals according to their host tree species and examined divergence on a locus‐by‐locus basis to assess whether specific genomic regions are associated with HAD. A total of 15 BayeScan runs were completed for each of these pairs. No significant *F*_ST_ outliers were detected using the FDR criterion (*q*‐value threshold of 0.05). Minimum and maximum *F*_ST_ values for individual loci for each pairwise comparison, as well as overall *F*_ST_ (Weir & Cockerham, 1984), are reported in Table 2.

**TABLE 2:** Minimum and maximum *F*_ST_ values from BayeScan analyses, estimated on a locus‐by‐locus basis between all sequenced individuals grouped by host association

Pairwise comparison	Minimum locus *F*_ST_ (BayeScan)	Maximum locus *F*_ST_ (BayeScan)	Overall *F*_ST_ (Weir & Cockerham, 1984)
Trembling aspen versus sugar maple	0.004	0.020	0.004
Trembling aspen versus red oak	0.006	0.011	0.004
Trembling aspen versus white birch	0.005	0.009	0.000
Sugar maple versus red oak	0.005	0.014	0.000
Sugar maple versus white birch	0.010	0.024	0.001
Red oak versus white birch	0.015	0.029	0.005

*Note*: BayeScan did not detect any significant outliers using a *q*‐value threshold of 0.05. Overall *F*_ST_ values were estimated between host association groups using the Weir and Cockerham (1984) method.

### 3.6 Environmental associations of individual loci (LFMM)

Across all eight environmental variables, LFMM identified a total of 64 loci with significant environmental associations (*p* < 0.05) after controlling for multiple tests with Bonferroni corrections applied to adjusted *p*‐values. Based on median |*z*|‐score, nine of these loci were most strongly associated with mean temperature of the warmest month, one with mean temperature of the coldest month, one with continentality, six with chilling degree days, five with growing degree days, two with extreme minimum temperature, 26 with mean summer precipitation, and 13 with length of the frost‐free period. For LFMM, visual inspection of adjusted *p*‐value histograms indicated that two latent factors (*K* = 2) adequately controlled for background population structure (Figure S3). In contrast, LFMM2 did not identify any significant genotype‐environment associations, even when using a more liberal FDR correction for multiple tests (Benjamini & Hochberg, 1995).

## 4 DISCUSSION

The aim of this study was to identify specific biotic and abiotic factors that underlie genomic differentiation in the forest tent caterpillar, *Malacosoma disstria*, a widespread and irruptive pest of numerous deciduous tree species in North America. Using a combination of landscape ecological modelling and high‐throughput DNA sequencing, we were able to quantify and compare the relative effects of geographic isolation (IBD + IBR), variation in environmental conditions (IBE), and host association (HAD as a special form of IBE) on population structure and genomic differentiation. Our analyses resolved that geographic isolation, spatial variation in summer temperatures, and host association are important factors contributing to genomic differentiation, demonstrating that multiple mechanisms that have not previously been considered are acting concurrently to structure intraspecific variation in this major forest pest. Evidence of these mechanisms was not apparent in genetic clustering analyses such as PCA and *structure*, highlighting the utility of landscape genomic methods in forest pest research.

### 4.1 Geographic isolation

Both RCM and dbRDA suggested significant IBD within *M. disstria*, with genomic differentiation among individuals increasing with their geographic separation (Figure 3, Table 1). In contrast, genomic differentiation was not related to resistance distances based on configurations of suitable habitat after controlling for IBD. Specifically, partial Mantel tests from RCMs show that both least‐cost and resistance distances were not significantly correlated with genetic distance after partialling out Euclidean distance (Table S4). Lack of support for IBR based on configurations of suitable habitat suggests that dispersal of *M. disstria* is likely agnostic with respect to habitat quality and resource availability. Although IBR is supported in a number of taxa (McRae & Beier, 2007; Sánchez‐Ramírez et al., 2018; Storfer et al., 2010; Thorpe et al., 2008; Wang et al., 2008, 2013), it has also been shown that low‐quality habitat within some animals' home ranges may not present significant barriers to movements during dispersal (e.g., Keeley et al., 2017; MacDonald et al., 2020). For insects, in particular, this result confers support for the dispersal machine hypothesis: although many insect species exhibit very specific habitat associations in their larval stage due to host plant dependencies, the adult stage (“dispersal machine”) often exhibits much greater vagility and broader habitat tolerances than larval life stages (MacDonald et al., 2020). Such characteristics are likely to facilitate long‐distance dispersal across heterogeneous landscapes that vary in habitat suitability, resulting in patterns of genomic differentiation that align more closely with IBD than IBR.

Another alternative hypothesis to IBD is that dispersal is primarily affected by dominant trade winds (Gatehouse, 1997). While *M. disstria* has relatively limited to moderate flight capacity (Fitzgerald, 1995; Strubel, 1970), strong weather fronts have been shown to transport large numbers of individuals over hundreds of kilometers (Brown, 1965). Genetic differentiation between local larvae and adults collected in traps that sample dispersers have been demonstrated in another forest pest species, *Choristoneura fumiferana*, with high dispersal rates related to prevailing winds (James et al., 2015; Nelson et al., 2022). Studies addressing genomic variation within *M. disstria* at broader spatial scales and across multiple time periods will be required to assess whether directionally biased gene flow, indicative of wind‐driven dispersal, exists across the species' range.

### 4.2 Environmental isolation in *M. disstria*

We observed considerable support for effects of IBE in *M. disstria* in both RCM and dbRDA, which identified various environmental distances that were positively correlated with genomic differentiation after controlling for other factors. Mechanistically, IBE can facilitate genomic differentiation within a species by moderating gene flow in three principal ways: (1) reduced tendency of individuals to disperse across environmental/ecological gradients (Edelaar & Bolnick, 2012); (2) reduced fitness of individuals that have dispersed across environmental/ecological gradients (Hendry, 2004; Wright, 1943); or (3) reduced fitness of individuals that are genetically intermediate between populations adapted for different niches (MacDonald et al., 2020). For any of these three mechanisms to affect gene flow, local adaptation to environmental conditions must first be present.

Our RCM analysis resolved that differences in summer temperatures and length of the growing season were significantly correlated with genetic distance among individuals after controlling for other factors (Figure 3). Like most other insects, *M. disstria* is ectothermic and individuals' growth, development, and reproductive fitness are closely tied to temperature. Larval growth and survival specifically have been documented to be significantly affected by spring and summer temperatures (Hodson, 1941; Levesque et al., 2002; Raske, 1975; Wetzel et al., 1973). Significant clinal or regional differences have also been detected in temperature‐associated functional traits in *M. disstria*, including spring larval emergence, cold tolerance, female resource allocation, and phenological synchrony (Lorimer, 1979; Mattson & Erickson, 1978; Parry et al., 2001; Uelmen, Duman, et al., 2016; Uelmen, Lindroth, et al., 2016). Spatial variation in temperature may therefore impose selective forces on *M. disstria*, whether directly via emergence and development or indirectly through synchrony with their host's phenology, providing opportunity for the evolution of regionally adapted populations.

Significant relationships between environmental distances and genomic differentiation suggest that local adaptation to environmental conditions is an important mechanism limiting gene flow and promoting genomic differentiation within *M. disstria*. Identifying specific genomic regions implicated in this local adaptation using genotype‐environment associations is an important subsequent step to understanding determinants of heritable variation within the species (Rellstab et al., 2015; Wagner & Fortin, 2013). However, our inferences varied substantially between two genotype‐environment association analyses that we employed. LFMM identified a total of 64 SNPs that were significantly associated with variation in environmental conditions, while the more conservative model implemented by LFMM2 did not detect any significant associations. Using empirical data as well as simulations, LFMM has been shown to exhibit greater power to resolve significant genotype‐environment associations for small datasets (e.g., those produced using reduced‐representation sequencing), both when the specified value of *K* is low and when population structure is continuous (Luo et al., 2021). However, LFMM may also have a higher false discovery rate than LFMM2 under these circumstances. Therefore, we cannot be sure whether the difference in our results between LFMM and LFMM2 is due to a higher false discovery rate of LFMM or reduced power of LFMM2. We have observed a similar difference in results between LFMM and LFMM2 when investigating genotype‐environment associations in the spruce budworm species complex (*Choristoneura fumiferana* and *C. occidentalis*; Nelson et al., 2022, and unpublished data). Ultimately, whole‐genome sequence data paired with an annotated reference genome will be needed to definitively identify specific genomic regions that are under ecologically mediated selection within the species. It is also important to consider that irruptive population dynamics of *M. disstria* may also lead to a high false discovery rate of loci under selection. Species, like *M. disstria*, with relatively weak dispersal abilities that are sampled early in their range expansion following outbreaks are particularly at risk of having neutral loci identified as adaptive (Mayrand et al., 2019). Thus, identification of specific loci under selection must be approached with caution.

### 4.3 Host‐associated differentiation

Host‐associated differentiation is considered a classic model of ecologically divergent selection and is a key evolutionary factor associated with diversification of phytophagous insects (Doellman & Feder, 2019; Drès & Mallet, 2002; Driscoe et al., 2019; Forbes et al., 2017; Funk et al., 2002; Leung & Beukeboom, 2021; Mackintosh et al., 2019; Medina, 2017; Peccoud et al., 2009; Vertacnik & Linnen, 2017). Both RCM and dbRDA identified that genomic differentiation within *M. disstria* is significantly related to host association after controlling for other factors. Although *F*_ST_ values among host‐associated groups are low and the proportion of overall genomic differentiation explained by host association is small, significant effects observed in RCM and dbRDA represent convincing molecular evidence of host‐associated adaptation in *M. disstria*. Importantly, these results align with previous documentation of reduced performance of larvae moved between natal host species. For example, in a series of reciprocal transplant experiments, Parry and Goyer (2004) demonstrated that *M. disstria* larvae had higher growth rate, pupal mass, and increased survival on their ovipositional host species than on alternate larval hosts across a latitudinal gradient of sites. Other *M. disstria* larval transplant experiments have given similar results (e.g., Nicol et al., 1997; Trudeau et al., 2010). Furthermore, *M. disstria* larvae have documented sensitivities to variation in host plant chemistry (Barbehenn & Martin, 1994; Hemming & Lindroth, 2000; Lindroth & Bloomer, 1991). Therefore, selection related to host association is expected if detoxification has a genetic basis in the species (e.g., Birnbaum & Abbot, 2020; Cohen et al., 1992; Orsucci et al., 2018). Additionally, despite extensive study of *M. disstria* larval performance, there has been little work on female host preference. Female *M. disstria* lay a single egg band on a host plant (Schowalter, 2017) and show limited dispersal (Fitzgerald, 1995; but cases of significant weather‐mediated dispersal events are known, Brown, 1965). Female oviposition choice has significant fitness consequences for larvae (Noseworthy & Despland, 2006) which are amplified by their gregarious behavior as early instars (Despland, 2013) and make it plausible for regional host preferences to evolve.

Despite the significant relationship between genomic differentiation and host association identified in RCM and dbRDA, we did not identify any loci with elevated *F*_ST_ values indicative of strong divergent selection. This may be due to the relatively small proportion of the total genome that is sequenced with ddRADseq, which may have missed localized “islands” of genomic divergence (Funk et al., 2021; Lowry et al., 2017; Riesch et al., 2017; Tiffin & Ross‐Ibarra, 2014). Additionally, genomic regions that appear neutral may still be under selection if selection acts upon highly polygenic traits, such that signatures of selection on individual genomic regions are too small to be detected (Balkenhol et al., 2017). Genomic architectures of complex traits, such as those related to host association, are increasingly recognized as polygenic in nature and may be associated with genomic variants besides single nucleotide substitutions (Allio et al., 2021; Boyle et al., 2017; Doellman & Feder, 2019; Gompert et al., 2015, 2022; Sella & Barton, 2019; Vertacnik & Linnen, 2017). For example, structural rearrangements, gene amplifications, and transposable element insertions underpin an adaptive host shift in the aphid pest *Myzus persicae* (Singh et al., 2020), which encompassed both widespread and localized mutational events. In the *Chloridea* spp. pest complex, the architecture of host association was spread across half of its 31 chromosomes and was linked to multiple traits including survival, feeding efficiency, and development (Oppenheim et al., 2018). Polygenic genomic architecture has also been shown in other adaptive traits such as eclosion time in *Rhagoletis* flies (Doellman et al., 2019; Meyers et al., 2020) and host resistance in *Callosobruchus maculatus* (Messina et al., 2021). Widespread genomic changes in a large number of genes have also been associated with host shifts and adaptive radiation in swallowtail butterfly lineages (Allio et al., 2021).

Although specific genomic regions under selection were not identified, results of RCM and dbRDA suggest that genomic differentiation within *M. disstria* in Eastern Canada is significantly related to both host association and variation in environmental conditions, but with evolutionary divergences insufficient for reproductive isolation and discrete population clustering. A chromosome‐level genome assembly paired with whole‐genome resequencing of individuals spanning the species' range will be required to understand the full extent of this differentiation and effectively search for localized islands of genomic divergence, structural rearrangements, gene amplifications, and transposable element insertions.

### 4.4 Applications to future research

This study demonstrates that geographic isolation, spatial variation in environmental conditions, and host association are all important factors underlying genomic differentiation in *M. disstria*. Evidence of ecologically mediated adaptation suggests that different populations, both sympatric and allopatric, have differential tolerances for similar environmental and ecological conditions. Whether emerging ecotypes or host races currently exist or are evolving across the range of *M. disstria* is not yet clear, but may explain significant regional variation in the duration and synchronicity of outbreaks, both in this species (Cooke & Roland, 2000; Roland, 1993; Wood et al., 2010) and other forest pests (Larroque et al., 2019; Nelson et al., 2022). Specific mechanisms linking genomic variation and selection to *M. disstria* outbreaks remain unexplored, but hold promise for understanding and predicting population dynamics (Saccheri & Hanski, 2006; Sinervo et al., 2000). If distinct host races exist at broad spatial scales, a viable option would be to partition outbreak models by host association, either by modelling groups separately or including group covariates. Furthermore, genomic evidence of local adaptation across environmental gradients may be integrated into ecological niche models to better map configurations of suitable habitat and predict the spatial extent of future outbreaks. Specifically, we have highlighted significant effects of summer temperatures and length of the growing season on genomic differentiation in *M. disstria*, which will help outbreak modelling efforts focus on a smaller subset of environmental parameters to explain regional variation in outbreak duration and synchronicity. This is particularly relevant for northern populations, which are expected to experience rapid shifts in local environmental conditions within the coming decades (Cooke & Roland, 2018; Schwartzberg et al., 2014). Finally, identifying specific genomic regions implicated in local adaptation will be an important next step to understanding specific functional traits that are subjected to environmental and host‐associated selection and mapping their distribution across the species' range.

## FUNDING INFORMATION

This work was supported by a Natural Sciences and Engineering Research Council (NSERC) Discovery Grant to F.A.H.S. (RGPIN‐2018‐04920), an NSERC Alexander Graham Bell Canada Graduate Scholarship – Doctoral (CGS‐D) and UCLA La Kretz Center for California Conservation Science Postdoctoral Fellowship to Z.G.M., and funding from Natural Resources Canada to A.D.R.

## CONFLICT OF INTEREST

The authors have no conflicts of interest to declare.

## Supporting information


Appendix S1

## Data Availability

DNA sequences in fastq format: GenBank accessions: SAMN30122098 – SAMN30122201; NCBI SRA: PRJNA865664. Sampling locations/metadata for sequenced individuals: Table S2.
