# Supplementary material for: Host association, environment, and geography underlie genomic differentiation in a major forest pest
Source: Evol Appl. 2022 Sep 23;15(11):1749–65. doi: 10.1111/eva.13466 (PMC9679251; doi:10.1111/eva.13466)
Supplement: Supplementary file 1 — Appendix S1 [file EVA-15-1749-s001.docx]

Supporting Information for:

Host association, environment, and geography underlie genomic differentiation in a major forest pest

Zachary G. MacDonald, Kyle Snape, Amanda D. Roe, Felix A. H. Sperling

Figure S1. Structure analyses including all 104 sequenced individuals indicated an optimal *K*-value of *K* = 2, using a) the Δ*K* method (Evanno et al. 2005) and b) the rate of change in the likelihood of *K* across *K* = 1:10 (Pritchard et al., 2000).

Figure S2. Structure admixture plots for *K* = 2:5. Within each admixture plot, individuals are sorted according to host association and then by increasing latitude.

Figure S3. Adjusted *P*-values for each environmental variable used in latent factor mixed modelling (LFMM). Adjusted *P*-values were estimated using the genomic inflation factor (λ) procedure described by Devlin & Roeder (1999). Distributions are relatively flat with a peak near zero, indicating that the selected number of latent factors (*K* = 2) adequately controlled for potentially confounding effects of spatial genetic structure.

Table S1. Sampling site details and outbreak conditions based on defoliation surveys conducted by provincial agencies. Number of egg bands and trees indicated when provenance is known. Defoliation status was defined based on four classes of defoliation used in provincial aerial surveys. Change in defoliation indicates whether the total area of defoliation increased or decreased in 2018 relative to 2017, and the years of growth indicates the number of years of increasing defoliation within a management district prior to 2018.

| Locality | District | Prov | Date | Number of Egg Bands | Number of Trees | Host Tree Species | Defoliation Status | Change in Defoliation | Years of Growth |
| --- | --- | --- | --- | --- | --- | --- | --- | --- | --- |
| Bancroft | Bancroft | ON | May, 2018 | 4 | 4 | *Acer saccharum* | Moderate-Severe | + | 3yr |
| Elliot Lake | Sudbury | ON | May, 2018 | 9 | 9 | *Populus tremuloides* | Moderate-Severe | + | 4yr |
| Foots Bay | Parry Sound | ON | May, 2018 | mixed | unknown | *Populus tremuloides* | Moderate-Severe | - | 1yr |
| Hearst | Hearst | ON | May, 2018 | mixed | unknown | *Populus tremuloides* | Moderate-Severe | - | 4yr |
| Kapuskasing | Hearst | ON | May, 2018 | mixed | unknown | *Populus tremuloides* | Moderate-Severe | - | 4yr |
| Killarny | Sudbury | ON | May, 2018 | mixed | unknown | *Populus tremuloides* | Moderate-Severe | + | 4yr |
| Killarny | Sudbury | ON | May, 2018 | mixed | unknown | *Betula papyrifera* | Moderate-Severe | + | 4yr |
| Lac Duparquet | Abitibi-Témiscamigue | QC | June, 2018 | mixed | unknown | *Populus tremuloides* | None | - | 4yr |
| Lanark | Kemptville | ON | June, 2018 | 5 | 5 | *Populus tremuloides* | Moderate-Severe | + | 3yr |
| Lanark | Kemptville | ON | June, 2018 | 4 | 4 | *Acer saccharum* | Moderate-Severe | + | 3yr |
| Latchford | North Bay | ON | May, 2018 | 4 | 4 | *Populus tremuloides* | Moderate-Severe | - | 2yr |
| Little Current | Sudbury | ON | May, 2018 | 3 | 3 | *Populus tremuloides* | Moderate-Severe | + | 4yr |
| Marten River | North Bay | ON | May, 2018 | mixed | unknown | *Populus tremuloides* | Moderate-Severe | - | 2yr |
| Montebello | Outaouais | QC | June, 2018 | mixed | unknown | *Acer saccharum* | Light-Moderate | + | 2yr |
| Nairn | Sudbury | ON | May, 2018 | 10 | 10 | *Populus tremuloides* | Moderate-Severe | + | 4yr |
| Oak Shores | Peterborough | ON | May, 2018 | mixed | unknown | *Quercus rubra* | Moderate-Severe | + | 3yr |
| Parry Sound | Parry Sound | ON | May, 2018 | 4 | 4 | *Acer saccharum* | Moderate-Severe | - | 1yr |
| Rabbit Lake | North Bay | ON | May, 2018 | 5 | 5 | *Populus tremuloides* | Moderate-Severe | - | 2yr |
| Sault Ste Marie | Sault Ste. Marie | ON | June, 2018 | 17 | 17 | *Populus tremuloides* | Moderate-Severe | + | 3yr |
| Sault Ste Marie | Sault Ste. Marie | ON | June, 2018 | 8 | 8 | *Acer saccharum* | Moderate-Severe | + | 3yr |
| Sault Ste Marie | Sault Ste. Marie | ON | June, 2018 | 7 | 7 | *Quercus rubra* | Moderate-Severe | + | 3yr |
| St. Joseph Island | Sault Ste. Marie | ON | June, 2018 | 9 | 9 | *Populus tremuloides* | Moderate-Severe | + | 3yr |
| St. Joseph Island | Sault Ste. Marie | ON | June, 2018 | 8 | 8 | *Acer saccharum* | Moderate-Severe | + | 3yr |
| Wharencliffe | Sault Ste. Marie | ON | May, 2018 | 1 | 1 | *Acer saccharum* | Moderate-Severe | + | 3yr |

*District: management/administrative region for provincial forest agencies; Number of Egg Bands: number of distinct egg bands sampled, mixed indicates that egg bands were shipped in a group and provenance of larvae could not be determined; Number of Trees: number of distinct trees that egg bands were collected from, unknown indicate total number of trees could not be determined; Defoliation Status: four defoliation classes defined by provincial agency (None, Light, Moderate, Severe); Change in Defoliation: increasing defoliation (+), decreasing defoliation (-) based on change in total area defoliated in 2017 vs 2018 within a management district; Years of Growth: number of years with increasing defoliation within a management district.*

Table S2. Metadata for *Malacosoma disstria* specimens used in landscape genomic analyses.

| Specimen ID | Prov | Locality | Latitude | Longitude | Collector | Date | Host Tree Species | Life Stage |
| --- | --- | --- | --- | --- | --- | --- | --- | --- |
| KS001 | ON | Lanark | 44.820 | -76.450 | Christi Jaeger | June 5, 2018 | *Acer saccharum* | larvae |
| KS006 | ON | Nairn | 46.302 | -81.678 | Ariel Ilic | May 8, 2018 | *Populus tremuloides* | larvae |
| KS011 | ON | Elliot | 46.339 | -82.542 | Mike Francis | May 9, 2018 | *Populus tremuloides* | larvae |
| KS012 | ON | Foots Bay | 45.166 | -79.760 | Ariel Ilic | May, 2018 | *Populus tremuloides* | larvae |
| KS013 | ON | Rabbit Lake | 46.930 | -79.726 | Chris McVeety | May 11, 2018 | *Populus tremuloides* | larvae |
| KS015 | ON | Oak Shores | 44.588 | -78.428 | Vanessa Chaimbrone | May 29, 2018 | *Quercus rubra* | larvae |
| KS016 | ON | Nairn | 46.302 | -81.678 | Ariel Ilic | May 8, 2018 | *Populus tremuloides* | larvae |
| KS017 | ON | Elliot | 46.339 | -82.542 | Mike Francis | May 9, 2018 | *Populus tremuloides* | larvae |
| KS018 | ON | Rabbit Lake | 46.930 | -79.726 | Chris McVeety | May 11, 2018 | *Populus tremuloides* | larvae |
| KS019 | ON | Lanark | 44.820 | -76.450 | Christi Jaeger | June 5, 2018 | *Populus tremuloides* | larvae |
| KS022 | ON | Lanark | 44.820 | -76.450 | Christi Jaeger | June 5, 2018 | *Acer saccharum* | larvae |
| KS025 | ON | Lanark | 44.820 | -76.450 | Christi Jaeger | June 5, 2018 | *Populus tremuloides* | larvae |
| KS027 | ON | Lanark | 44.820 | -76.450 | Christi Jaeger | June 5, 2018 | *Acer saccharum* | larvae |
| KS034 | ON | Lanark | 44.820 | -76.450 | Christi Jaeger | June 5, 2018 | *Acer saccharum* | larvae |
| KS045 | ON | Lanark | 44.820 | -76.450 | Christi Jaeger | June 5, 2018 | *Populus tremuloides* | larvae |
| KS050 | ON | Foots Bay | 45.166 | -79.760 | Ariel Ilic | Ma,y 2018 | *Populus tremuloides* | larvae |
| KS051 | QUE | Montebello | 45.700 | -74.800 | Anne-Sophie Caron | June, 2018 | *Acer saccharum* | larvae |
| KS057 | ON | Rabbit Lake | 46.930 | -79.726 | Chris McVeety | May 11, 2018 | *Populus tremuloides* | larvae |
| KS058 | ON | Elliot | 46.339 | -82.542 | Mike Francis | May 9, 2018 | *Populus tremuloides* | larvae |
| KS064 | ON | Sault Ste Marie | 46.510 | -84.267 | Amanda Roe, Kyle Snape | June 7, 2018 | *Populus tremuloides* | adult |
| KS068 | ON | Sault Ste Marie | 46.510 | -84.267 | Amanda Roe, Kyle Snape | June 7, 2018 | *Populus tremuloides* | larvae |
| KS069 | ON | Hearst | 49.731 | -83.913 | Lia Fricano | May 9, 2018 | *Populus tremuloides* | larvae |
| KS070 | ON | Sault Ste Marie | 46.510 | -84.267 | Amanda Roe, Kyle Snape | June 7, 2018 | *Populus tremuloides* | adult |
| KS072 | ON | Nairn | 46.302 | -81.678 | Ariel Ilic | May 8, 2018 | *Populus tremuloides* | larvae |
| KS074 | ON | Hearst | 49.731 | -83.913 | Lia Fricano | May 9, 2018 | *Populus tremuloides* | larvae |
| KS077 | ON | Latchford | 47.333 | -79.810 | Chris McVeety | May 2018 | *Populus tremuloides* | larvae |
| KS078 | ON | Sault Ste Marie | 46.510 | -84.267 | Amanda Roe, Kyle Snape | June 7, 2018 | *Populus tremuloides* | adult |
| KS079 | ON | Sault Ste Marie | 46.508 | -84.302 | Amanda Roe, Kyle Snape | June 7, 2018 | *Quercus rubra* | adult |
| KS080 | ON | Sault Ste Marie | 46.510 | -84.267 | Amanda Roe, Kyle Snape | June 7, 2018 | *Populus tremuloides* | adult |
| KS081 | ON | Sault Ste Marie | 46.510 | -84.267 | Amanda Roe, Kyle Snape | June 7, 2018 | *Populus tremuloides* | adult |
| KS082 | ON | Sault Ste Marie | 46.506 | -84.306 | Amanda Roe, Kyle Snape | June 7, 2018 | *Acer saccharum* | adult |
| KS083 | ON | Foots Bay | 45.166 | -79.760 | Ariel Ilic | May 2018 | *Populus tremuloides* | larvae |
| KS085 | ON | Killarney | 46.011 | -81.401 | Ariel Ilic | May 2018 | *Betula papyrifera* | larvae |
| KS087 | ON | Sault Ste Marie | 46.510 | -84.267 | Amanda Roe, Kyle Snape | June 7, 2018 | *Populus tremuloides* | larvae |
| KS088 | ON | St Joseph Island | 46.192 | -84.042 | Kyle Snape, Reshma Jose | June 3, 2018 | *Acer saccharum* | adult |
| KS089 | ON | Sault Ste Marie | 46.510 | -84.267 | Amanda Roe, Kyle Snape | June 7, 2018 | *Populus tremuloides* | adult |
| KS090 | ON | St Joseph Island | 46.192 | -84.042 | Kyle Snape, Reshma Jose | June 3, 2018 | *Acer saccharum* | adult |
| KS091 | ON | Sault Ste Marie | 46.510 | -84.267 | Amanda Roe, Kyle Snape | June 7, 2018 | *Populus tremuloides* | adult |
| KS095 | ON | Latchford | 47.333 | -79.810 | Chris McVeety | May 2018 | *Populus tremuloides* | larvae |
| KS096 | ON | Marten River | 46.668 | -79.728 | Chris McVeety | May 2018 | *Populus tremuloides* | larvae |
| KS097 | ON | Sault Ste Marie | 46.510 | -84.267 | Amanda Roe, Kyle Snape | June 7, 2018 | *Populus tremuloides* | adult |
| KS101 | ON | Little Current | 45.879 | -81.899 | Ariel Ilic | May 2018 | *Populus tremuloides* | adult |
| KS104 | ON | Sault Ste Marie | 46.508 | -84.302 | Amanda Roe, Kyle Snape | June 7, 2018 | *Quercus rubra* | adult |
| KS106 | ON | Kapuskasing | 49.644 | -82.302 | Lia Fricano | May 8, 2018 | *Populus tremuloides* | larvae |
| KS107 | ON | St Joseph Island | 46.192 | -84.042 | Kyle Snape, Reshma Jose | June 3, 2018 | *Populus tremuloides* | adult |
| KS112 | ON | Sault Ste Marie | 46.510 | -84.267 | Amanda Roe, Kyle Snape | June 7, 2018 | *Populus tremuloides* | adult |
| KS113 | ON | Marten River | 46.668 | -79.728 | Chris McVeety | May 2018 | *Populus tremuloides* | adult |
| KS115 | ON | St Joseph Island | 46.192 | -84.042 | Kyle Snape, Reshma Jose | June 3, 2018 | *Populus tremuloides* | adult |
| KS116 | ON | Sault Ste Marie | 46.508 | -84.302 | Amanda Roe, Kyle Snape | June 7, 2018 | *Quercus rubra* | adult |
| KS120 | ON | Oak Shores | 44.588 | -78.428 | Vanessa Chaimbrone | May 29, 2018 | *Quercus rubra* | adult |
| KS124 | ON | Sault Ste Marie | 46.506 | -84.306 | Amanda Roe, Kyle Snape | June 7, 2018 | *Acer saccharum* | adult |
| KS126 | ON | Sault Ste Marie | 46.506 | -84.306 | Amanda Roe, Kyle Snape | June 7, 2018 | *Acer saccharum* | adult |
| KS129 | ON | St Joseph Island | 46.192 | -84.042 | Kyle Snape, Reshma Jose | June 3, 2018 | *Populus tremuloides* | adult |
| KS130 | ON | Elliot | 46.339 | -82.542 | Mike Francis | May 9, 2018 | *Populus tremuloides* | larvae |
| KS132 | ON | Sault Ste Marie | 46.510 | -84.267 | Amanda Roe, Kyle Snape | June 7, 2018 | *Populus tremuloides* | adult |
| KS133 | ON | Sault Ste Marie | 46.510 | -84.267 | Amanda Roe, Kyle Snape | June 7, 2018 | *Populus tremuloides* | adult |
| KS136 | ON | St Joseph Island | 46.192 | -84.042 | Kyle Snape, Reshma Jose | June 3, 2018 | *Populus tremuloides* | adult |
| KS138 | ON | Marten River | 46.668 | -79.728 | Chris McVeety | May 2018 | *Populus tremuloides* | larvae |
| KS139 | ON | Rabbit Lake | 46.930 | -79.726 | Chris McVeety | May 11, 2018 | *Populus tremuloides* | larvae |
| KS141 | ON | Sault Ste Marie | 46.506 | -84.306 | Amanda Roe, Kyle Snape | June 7, 2018 | *Acer saccharum* | adult |
| KS143 | ON | Rabbit Lake | 46.930 | -79.726 | Chris McVeety | May 11, 2018 | *Populus tremuloides* | larvae |
| KS144 | ON | Foots Bay | 45.166 | -79.760 | Ariel Ilic | May 2018 | *Populus tremuloides* | adult |
| KS146 | ON | Sault Ste Marie | 46.510 | -84.267 | Amanda Roe, Kyle Snape | June 7, 2018 | *Populus tremuloides* | larvae |
| KS147 | QUE | Montebello | 45.700 | -74.800 | Anne-Sophie Caron | June 2018 | *Acer saccharum* | larvae |
| KS148 | ON | Latchford | 47.333 | -79.810 | Chris McVeety | May 2018 | *Populus tremuloides* | larvae |
| KS154 | ON | Nairn | 46.302 | -81.678 | Ariel Ilic | May 8, 2018 | *Populus tremuloides* | larvae |
| KS155 | ON | Hearst | 49.731 | -83.913 | Lia Fricano | May 9, 2018 | *Populus tremuloides* | larvae |
| KS157 | ON | Parry Sound | 45.378 | -80.044 | Ariel Ilic | May 9, 2018 | *Acer saccharum* | larvae |
| KS159 | ON | Nairn | 46.302 | -81.678 | Ariel Ilic | May 8, 2018 | *Populus tremuloides* | larvae |
| KS161 | ON | St Joseph Island | 46.192 | -84.042 | Kyle Snape, Reshma Jose | June 3, 2018 | *Acer saccharum* | adult |
| KS162 | ON | Hearst | 49.731 | -83.913 | Lia Fricano | May 9, 2018 | *Populus tremuloides* | larvae |
| KS163 | ON | Foots Bay | 45.166 | -79.760 | Ariel Ilic | May 2018 | *Populus tremuloides* | larvae |
| KS164 | ON | Kapuskasing | 49.644 | -82.302 | Lia Fricano | May 8, 2018 | *Populus tremuloides* | larvae |
| KS166 | ON | Elliot | 46.339 | -82.542 | Mike Francis | May 9, 2018 | *Populus tremuloides* | larvae |
| KS167 | ON | Kapuskasing | 49.644 | -82.302 | Lia Fricano | May 2018 | *Populus tremuloides* | larvae |
| KS169 | QUE | Montebello | 45.700 | -74.800 | Anne-Sophie Caron | June 2018 | *Acer saccharum* | larvae |
| KS170 | ON | St Joseph Island | 46.192 | -84.042 | Kyle Snape, Reshma Jose | June 3, 2018 | *Populus tremuloides* | adult |
| KS180 | ON | Rabbit Lake | 46.930 | -79.726 | Chris McVeety | May 11, 2018 | *Populus tremuloides* | larvae |
| KS183 | ON | Sault Ste Marie | 46.510 | -84.267 | Amanda Roe, Kyle Snape | June 7, 2018 | *Populus tremuloides* | adult |
| KS184 | ON | St Joseph Island | 46.192 | -84.042 | Kyle Snape, Reshma Jose | June 3, 2018 | *Populus tremuloides* | adult |
| KS185 | ON | Nairn | 46.302 | -81.678 | Ariel Ilic | May 8, 2018 | *Populus tremuloides* | larvae |
| KS186 | ON | Killarney | 46.011 | -81.401 | Ariel Ilic | May 2018 | *Betula papyrifera* | larvae |
| KS188 | ON | Marten River | 46.668 | -79.728 | Chris McVeety | May 2018 | *Populus tremuloides* | larvae |
| KS190 | ON | Kapuskasing | 49.644 | -82.302 | Lia Fricano | May 8, 2018 | *Populus tremuloides* | larvae |
| KS192 | ON | Sault Ste Marie | 46.510 | -84.267 | Amanda Roe, Kyle Snape | June 7, 2018 | *Populus tremuloides* | adult |
| KS194 | ON | Killarney | 46.011 | -81.401 | Ariel Ilic | May 2018 | *Betula papyrifera* | adult |
| KS196 | ON | Killarney | 46.011 | -81.401 | Ariel Ilic | May 2018 | *Betula papyrifera* | larvae |
| KS198 | ON | Sault Ste Marie | 46.506 | -84.306 | Amanda Roe, Kyle Snape | June 7, 2018 | *Acer saccharum* | adult |
| KS201 | ON | Marten River | 46.668 | -79.728 | Chris McVeety | May 2018 | *Populus tremuloides* | larvae |
| KS205 | ON | St Joseph Island | 46.192 | -84.042 | Kyle Snape, Reshma Jose | June 3, 2018 | *Acer saccharum* | adult |
| KS207 | ON | Hearst | 49.731 | -83.913 | Lia Fricano | May 9, 2018 | *Populus tremuloides* | larvae |
| KS210 | ON | Latchford | 47.333 | -79.810 | Chris McVeety | May 2018 | *Populus tremuloides* | larvae |
| KS213 | ON | St Joseph Island | 46.192 | -84.042 | Kyle Snape, Reshma Jose | June 3, 2018 | *Populus tremuloides* | adult |
| KS214 | ON | Wharncliffe | 46.538 | -83.437 | Mike Francis | May 2018 | *Acer saccharum* | larvae |
| KS215 | ON | Sault Ste Marie | 46.508 | -84.302 | Amanda Roe, Kyle Snape | June 7, 2018 | *Quercus rubra* | adult |
| KS216 | ON | Sault Ste Marie | 46.506 | -84.306 | Amanda Roe, Kyle Snape | June 7, 2018 | *Acer saccharum* | adult |
| KS217 | ON | Sault Ste Marie | 46.506 | -84.306 | Amanda Roe, Kyle Snape | June 7, 2018 | *Acer saccharum* | adult |
| KS218 | ON | Little Current | 45.879 | -81.899 | Ariel Ilic | May 2018 | *Populus tremuloides* | larvae |
| KS219 | ON | Foots Bay | 45.166 | -79.760 | Ariel Ilic | May 2018 | *Populus tremuloides* | larvae |
| KS228 | ON | Bancroft | 44.887 | -77.747 | Vanessa Chaimbrone | May 16, 2018 | *Acer saccharum* | larvae |
| KS229 | QUE | Duparquet | 48.500 | -79.200 | Joshua Jarry | June 2018 | *Populus tremuloides* | larvae |
| KS233 | QUE | Montebello | 45.700 | -74.800 | Anne-Sophie Caron | June 2018 | *Acer saccharum* | larvae |
| KS234 | ON | St Joseph Island | 46.192 | -84.042 | Kyle Snape, Reshma Jose | June 3, 2018 | *Acer saccharum* | adult |
| KS241 | ON | Bancroft | 44.887 | -77.747 | Vanessa Chaimbrone | May 16, 2018 | *Acer saccharum* | larvae |

Table S3. Results of reciprocal causal modelling (RCM) that assess correlates of genomic differentiation (Euclidean genetic distance) in *Malacosoma disstria*. Values in each cell represent results of *R_PM-A_* - *R_PM-B_*. Rows and columns contain the focal and alternative variables, respectively, for partial Mantel test A within each reciprocal model. This table should be interpreted by rows and not columns; variables with more positive values in their rows are the strongest correlates of genomic differentiation after partialling out relationships with alternative variables.

|  | Euclidean distance | least-cost distance | resistance distance | mean temp. warmest month | mean temp. coldest month | continentality | chilling degree days | growing degree days | extreme min. temp. | mean summer precip. | frost-free period | host association |
| --- | --- | --- | --- | --- | --- | --- | --- | --- | --- | --- | --- | --- |
| Euclidean distance | NA | 0.556 | 0.572 | 0.071 | 0.446 | 0.449 | 0.471 | 0.281 | 0.408 | 0.209 | 0.401 | -0.106 |
| least-cost distance | -0.556 | NA | 0.111 | -0.560 | -0.129 | -0.171 | -0.107 | -0.536 | -0.193 | -0.185 | -0.407 | -0.392 |
| resistance distance | -0.572 | -0.111 | NA | -0.593 | -0.165 | -0.197 | -0.147 | -0.551 | -0.231 | -0.205 | -0.456 | -0.400 |
| mean temp. warmest month | -0.071 | 0.560 | 0.593 | NA | 0.402 | 0.337 | 0.448 | 0.397 | 0.359 | 0.140 | 0.540 | -0.135 |
| mean temp. coldest month | -0.446 | 0.129 | 0.165 | -0.402 | NA | -0.142 | 0.156 | -0.373 | -0.309 | -0.196 | -0.208 | -0.355 |
| continentality | -0.449 | 0.171 | 0.197 | -0.337 | 0.142 | NA | 0.164 | -0.273 | 0.023 | -0.181 | -0.107 | -0.335 |
| chilling degree days | -0.471 | 0.107 | 0.147 | -0.448 | -0.156 | -0.164 | NA | -0.435 | -0.298 | -0.200 | -0.266 | -0.367 |
| growing degree days | -0.281 | 0.536 | 0.551 | -0.397 | 0.373 | 0.273 | 0.435 | NA | 0.313 | 0.041 | 0.444 | -0.220 |
| extreme min. temp. | -0.408 | 0.193 | 0.231 | -0.359 | 0.309 | -0.023 | 0.298 | -0.313 | NA | -0.166 | -0.144 | -0.338 |
| mean summer precip. | -0.209 | 0.185 | 0.205 | -0.140 | 0.196 | 0.181 | 0.200 | -0.041 | 0.166 | NA | 0.075 | -0.258 |
| frost-free period | -0.401 | 0.407 | 0.456 | -0.540 | 0.208 | 0.107 | 0.266 | -0.444 | 0.144 | -0.075 | NA | -0.319 |
| host association | 0.106 | 0.392 | 0.400 | 0.135 | 0.355 | 0.335 | 0.367 | 0.220 | 0.338 | 0.258 | 0.319 | NA |

Table S4. *P*-values of partial Mantel test A for each reciprocal causal model (RCM) presented in Table S2. Rows represent focal variables from partial Mantel test A; i.e., variables that were correlated with genomic differentiation (Euclidean genetic distance) after partialling out alternative variables shown in columns. This table should be interpreted by rows and not columns; variables with lower *P*-values in their rows are the strongest correlates of genomic differentiation after partialling out relationships with alternative variables.

|  | Euclidean distance | least-cost distance | resistance distance | mean temp. warmest month | mean temp. coldest month | continentality | chilling degree days | growing degree days | extreme min. temp. | mean summer precip. | frost-free period | host association |
| --- | --- | --- | --- | --- | --- | --- | --- | --- | --- | --- | --- | --- |
| Euclidean distance | NA | 0.001 | 0.001 | 0.064 | 0.001 | 0.001 | 0.001 | 0.003 | 0.001 | 0.014 | 0.001 | 0.120 |
| least-cost distance | 1.000 | NA | 0.374 | 1.000 | 0.974 | 0.984 | 0.959 | 0.999 | 0.985 | 0.996 | 1.000 | 0.994 |
| resistance distance | 1.000 | 0.896 | NA | 1.000 | 0.985 | 0.991 | 0.981 | 1.000 | 0.998 | 0.997 | 1.000 | 0.998 |
| mean temp. warmest month | 0.564 | 0.001 | 0.001 | NA | 0.007 | 0.039 | 0.004 | 0.001 | 0.020 | 0.190 | 0.001 | 0.313 |
| mean temp. coldest month | 1.000 | 0.586 | 0.458 | 1.000 | NA | 0.986 | 0.102 | 1.000 | 0.999 | 0.996 | 0.998 | 0.998 |
| continentality | 1.000 | 0.493 | 0.399 | 0.999 | 0.150 | 1.000 | 0.168 | 1.000 | 0.709 | 0.996 | 0.994 | 0.998 |
| chilling degree days | 1.000 | 0.580 | 0.491 | 1.000 | 0.955 | 0.985 | 1.000 | 1.000 | 0.999 | 0.994 | 1.000 | 0.996 |
| growing degree days | 0.975 | 0.001 | 0.001 | 0.998 | 0.030 | 0.134 | 0.010 | 1.000 | 0.073 | 0.620 | 0.002 | 0.718 |
| extreme min. temp. | 1.000 | 0.291 | 0.188 | 0.999 | 0.009 | 0.825 | 0.008 | 1.000 | NA | 0.993 | 0.995 | 0.989 |
| mean summer precip. | 0.952 | 0.867 | 0.833 | 0.900 | 0.568 | 0.484 | 0.581 | 0.878 | 0.604 | NA | 0.833 | 0.920 |
| frost-free period | 0.997 | 0.002 | 0.001 | 1.000 | 0.275 | 0.664 | 0.106 | 1.000 | 0.457 | 0.960 | 1.000 | 0.963 |
| host association | 0.002 | 0.005 | 0.007 | 0.005 | 0.006 | 0.003 | 0.006 | 0.006 | 0.006 | 0.003 | 0.002 | 1.000 |

Table S5. Positive eigenvectors (dbMEM variables) identified by principal coordinates of neighbour matrices analysis applied to Euclidean distances among sequenced individuals. Significant dbMEMs were identified using permutational ANOVA applied to dbRDA with genetic distance as the response matrix. Significant dbMEMS were then incorporated into our final dbRDA (Table 1).

|  | Df | SumOfSqs | F | Pr(>F) |
| --- | --- | --- | --- | --- |
| dbMEM 1 | 1 | 0.587 | 1.3661 | 0.001*** |
| dbMEM 2 | 1 | 0.475 | 1.1057 | 0.012* |
| dbMEM 3 | 1 | 0.723 | 1.6835 | 0.001*** |
| dbMEM 4 | 1 | 0.439 | 1.0214 | 0.27 |
| dbMEM 5 | 1 | 0.427 | 0.9934 | 0.58 |
| dbMEM 6 | 1 | 0.458 | 1.0673 | 0.039* |
| dbMEM 7 | 1 | 0.423 | 0.9852 | 0.652 |
| dbMEM 8 | 1 | 0.435 | 1.0136 | 0.316 |
| dbMEM 9 | 1 | 0.415 | 0.967 | 0.829 |
| dbMEM 10 | 1 | 0.421 | 0.9795 | 0.681 |
| dbMEM 11 | 1 | 0.418 | 0.9731 | 0.751 |

Significance denoted by: * P < 0.05; ** P < 0.01; *** P < 0.001

**References**

Devlin, B., & Roeder, K. (1999). Genomic control for association studies. Biometrics, 55(4), 997-1004.

Evanno, G., Regnaut, S., & Goudet, J. (2005). Detecting the number of clusters of individuals using the software STRUCTURE: A simulation study. Molecular Ecology, 14(8), 2611-2620.

Pritchard, J. K., Stephens, M., & Donnelly, P. (2000). Inference of population structure using multilocus genotype data. Genetics, 155(2), 945-959.
